# Research on the Thermal Aging Performance of a GAP-Based Polyurethane Elastomer

**DOI:** 10.3390/polym16060795

**Published:** 2024-03-13

**Authors:** Chang Liu, Fengdan Zhu, Desheng Yang, Chaofei Bai, Xiaoqing Wang, Guoping Li, Yunjun Luo

**Affiliations:** 1 School of Materials Science & Engineering, Beijing Institute of Technology, Beijing 100081, China; changliujy@163.com (C.L.); zhufd163email@163.com (F.Z.); ydslww@163.com (D.Y.); superbcf@163.com (C.B.); yjluo@bit.edu.cn (Y.L.); 2 China Key Laboratory for Ministry of Education of High Energy Density Materials, Ministry of Education, Beijing 100081, China

**Keywords:** GAP elastomer, aging behavior, microstructure, mechanical properties

## Abstract

Glycidyl azide polymer (GAP)-based polyurethane is an ideal elastomeric matrix for high-energy, low-smoke, and insensitive solid propellants. As the skeleton structure of GAP propellants, changes in the structure and properties of GAP elastomers during aging lead to the deterioration of propellant performance (especially in relation to mechanical properties), which causes safety risks. A high-temperature-accelerated aging experiment (70 °C) on a GAP elastomer was conducted. The evolution of the microstructure of the GAP elastomer system was analyzed by Fourier-transform infrared spectroscopy (FTIR) and nuclear magnetic resonance spectroscopy (NMR), and variations in the macroscopic properties were analyzed by the hardness test and the uniaxial tensile test. The experimental results showed that thermal aging of the GAP elastomer is a coupled process of multiple chemical reactions. The azide groups, urethane groups, and ether bonds were the weak links in the network structure, breaking during the aging process, and the crosslinking density rose and then decreased. Macroscopic properties also showed segmented changes. The aging process was divided into three stages: post-curing (stage one); when the crosslinked network began to break (stage two), and when the crosslinked network was destroyed (stage three). Changes in the microstructure and macroscopic properties were consistent. This work is of great significance for exploring the aging mechanism of GAP propellants and extending their storage life.

## 1. Introduction

Various properties of solid propellants (especially mechanical properties) are inevitably degraded during prolonged storage due to the influence of the environment and the interaction between the components [1]; this is known as aging [2]. During the aging process, solid propellants may suffer from deformation, cracking, and other damage [3], which gradually fail to meet the use requirements and give rise to serious safety hazards, so aging performance is crucial for propellants. Research on the aging mechanism of solid propellants can provide a basis for predicting service life, thus avoiding accidents caused by propellants not being scrapped in time at the end of their service life. Ozupek [4] proposed a comprehensive propellant model, which can mathematically represent the mechanical changes during propellant aging and calculate the service life of the propellant through finite element analysis. Djalal [5] explored the thermal decomposition kinetics of aging propellants by DSC and predicted storage life by van’t Hoff’s equation. This is a common method to predict the service life of propellants according to the change in mechanical properties during aging [6]. In addition, anti-aging measures have been adopted to improve the storage life of propellants. Luciene [7] studied the effect of the antioxidant 2,6-di(tertbutyl)hydroxytoluene (BHT) on HTPB propellants during thermal aging and found that the reactivity of HTPB and the curing agent (IPDI) increased gradually with the consumption of BHT. The aging resistance coefficient of HTPB propellant can be improved by the anti-oxidant Flexzone 6-H, and thus the service life will be extended [8]. A large number of studies have shown that the performance changes in solid propellants during storage are closely related to the aging behavior of their polymer elastic matrix. The degree of crosslinking is an important link in facilitating the communication between chemical aging and aged mechanical parameters [9]. Chen [10] investigated the aging mechanism of PET elastomers and established a mechanical property deterioration model of propellants based on microstructure evolution. Thus, by analyzing the changes in the physical and chemical properties of the elastic matrix, it is possible to determine the key reactions and degradation mechanisms during the aging process, which allows us to reveal the aging mechanism [11] and extend the service life of the propellants.

The elastic matrix of the propellant is a network structure formed after the curing reaction between the binder system and the curing-agent system [12]. As the core composition and skeleton matrix of the propellants, the elastic matrix has a significant effect on mechanical properties during the aging process [13]. The aging mechanism is often studied from two aspects: the microstructure and the macroproperties [14] The aging mechanism of solid propellants varies with elastomers, so the mechanical properties changes in the aging process are also different. The hydroxy-terminated polybutadiene (HTPB) propellants are prone to oxidative crosslinking reaction in the aging process due to the skeleton having a large number of C=C unsaturated groups. As a result, the macroscopic properties change with increased maximum tensile strength and the maximum elongation decreases during the aging process [15,16]. The mechanical properties of polyether (NEPE) propellants containing a large amount of nitrate-based plasticizers deteriorate gradually during aging [17]. This is due to the nitrate-based plasticizers releasing nitrogen oxides and breaking the elastic matrix of the adhesive [18].

Glycidyl azide polymer (GAP) is a kind of polyether prepolymer with azide side groups [19]. The large number of azide side groups gives it the advantages of high energy, high density, good thermal stability, low sensibility, and low impulse [20,21], as well as good compatibility with nitrate- and azide energy-containing plasticizers. Therefore, the polyurethane high-energy elastomeric matrix prepared with GAP as a prepolymer is used in solid propellants, which can well satisfy the demand for the development of propellants with high energy, low sensibility, and low smoke [22]. GAP-based propellants exhibit unique mechanical changes during aging due to their elastomers [23], but the structural evolution and aging mechanism of pure GAP-based elastomers during thermal aging are still unclear.

In this work, GAP polyurethane elastomers were prepared by using GAP as the prepolymer and N-100 as the curing agent. A heat-aging experiment was carried out on GAP elastomers at 70 °C. Changes in the macroscopic properties and microstructure of elastomers during the heat-aging process were investigated, and the high-temperature aging characteristics and aging mechanism of GAP elastomers were analyzed from the point of view of microscopic evolution.

## 2. Materials and Methods

### 2.1. Materials

Glycidyl azide polymer (GAP, M_W_ = 3704 g/mol) was supplied by Liming Research Institute of Chemical Industry (Luoyang, China) and dried in a vacuum oven at 80 °C for 12 h to remove water before use.

The isocyanate curing agent N-100 (M_W_ = 744 g/mol) was supplied by Liming Research Institute of Chemical Industry (Luoyang, China), dried, and preserved by removing water.

### 2.2. Preparation of GAP Elastomer

**Figure 1:**
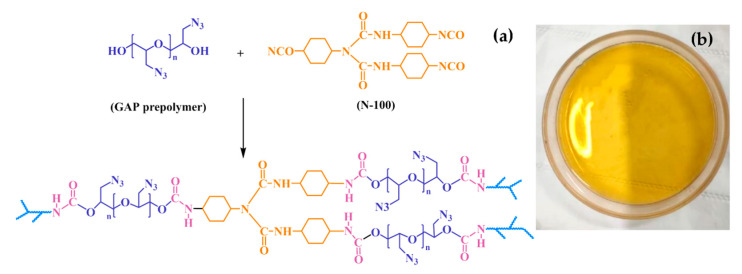
(**a**) Curing reaction mechanism. (**b**) Samples in the high-temperature-accelerated aging experiment.

The curing ratio of the fixed GAP elastomer was 1.4 (the reaction group equivalent ratio of N-100 to GAP, *R*_-NCO/-OH_). GAP and N-100 were weighed and mixed evenly, then poured into a preheated curing mold and put into a vacuum oven at 60 °C to remove air bubbles. After the surface of the sample was leveled and bubbles no longer emerged, it was removed and placed into a watertight constant-temperature incubator at 60 °C for 7 days to cure; the curing mechanism is shown in Figure 1a. The GAP elastomers were prepared as shown in Figure 1b.

### 2.3. Methods

#### 2.3.1. Thermal-Accelerated Aging Test

To ensure that the humidity during the aging process was less than 10% (RH < 10%), the experimental samples were packed in aluminum foil-sealed bags, and the high-temperature-accelerated aging experiments were conducted under a watertight constant-temperature incubator at 70 °C. Samples were taken out for the different number of aging days according to the needs. The removed samples were cut into desired shapes and placed in a desiccator to naturally cool to room temperature for testing. The time from sampling to testing was usually completed within 2 days, with a maximum of 7 days. The specimen was placed in a desiccator for proper storage during the period to be tested.

#### 2.3.2. Mechanical Characterization

Shore A (Peak Technology Ltd., Beijing, China) hardness tester was used to test the hardness of the samples with different aging times at room temperature. The thickness of the specimen was not less than 6 mm, the width of the specimen was not less than 25 mm, and the length of the specimen was not less than 40 mm. The surface-hardness value of the elastomers is the reading after 30 s of full contact between the needle of the hardness tester and the sample. Each sample was tested 5 times, and the average value was taken.

A uniaxial tensile testing machine (Shimadzu Corporation, Kyoto, Japan) was used to conduct room-temperature uniaxial tensile experiments on samples with different aging times at a tensile rate of 100 mm/min. The GAP elastomers were taken out at the predetermined storage time, and the GAP elastomers were prepared as dumbbell-shaped samples of 12 mm (neck length) × 2 mm (width) × 2 mm (thickness), which were placed in a desiccator and cooled naturally for one day and then subjected to stress release after the uniaxial tensile test. Each group consisted of 5 test samples, and the results were averaged.

#### 2.3.3. Thermal Characterization

Thermogravimetric infrared spectroscopy (TG-IR) was used to analyze the thermal decomposition gases of the unaged samples, which were particulate samples with a sample mass of 0.6~1 mg. Air was used as the protective and purge gas, with a total flow rate of 55 mL/min. The test temperature was 30~800 °C, with a temperature increase rate of 10 K/min. The MCT-type (Thermo Fisher Scientific, Shanghai, China) infrared detector was used to collect the infrared information of the decomposition gases, with an infrared resolution of 4 cm^−1^ and a wave number of 4000~500 cm^−1^. The TG-IR tests were performed on the Thermal Analyser (STA449F3) from NETZSCH-Gerätebau GmbH (Selb, Germany) and FTIR (Nicolet iS20) from Thermo Fisher Scientific (Shanghai, China), which were provided by eceshi (www.eceshi.com).

Thermogravimetric differential scanning calorimetry (TG-DSC) was used to test the samples with different aging times to calculate the activation energy. The sample mass was 0.6~1 mg of granular samples. Air or nitrogen was used as the protective and purging gas, with a total flow rate of 40 mL/min. The test temperature was 30~800 °C, and the temperature increase rate was 10 K/min. The tests were performed using the Thermal Analyser (TGA/DSC 3+) from Mettler Toledo Inc (Schwerzenbach, Switzerland).

#### 2.3.4. Chemical Characterization

The samples were tested during the aging process using a Fourier-transform infrared (FTIR) (Thermo Fisher Scientific, Shanghai, China) photometer with a scanning wave-number range of 4000–400 cm^−1^, a resolution of 4 cm^−1^, and 32 scans.

#### 2.3.5. Crosslinked Network Characteristics

A low-field NMR spectrometer (Niumag Corporation, Suzhou, China) was used to test the samples during the aging process by cutting the samples into 2 mm × 2 mm × 2 mm slices, mixing them well in NMR tubes, and placing them in a cuvette to keep them warm for 20 min and then testing them at an experimental temperature of 35 °C.

## 3. Results

### 3.1. The Thermal Decomposition Behavior of Unaged GAP Elastomers

**Figure 2:**
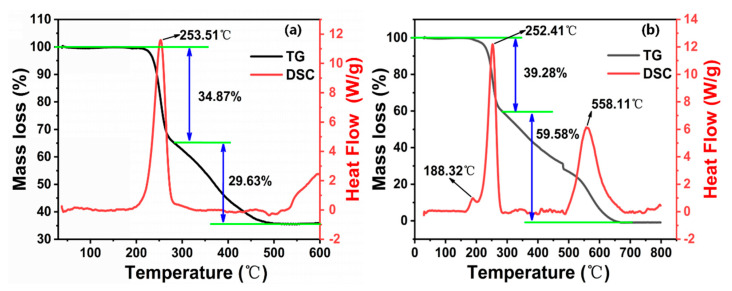
TG-DSC curves of unaged GAP elastomers under different atmospheres: (**a**) Nitrogen atmosphere; (**b**) Air atmosphere.

The study of the thermal decomposition of GAP elastomers is necessary to understand and analyze their thermally accelerated aging mechanism. By revealing the key reactions and products in the thermal decomposition process, we can gain insight into the causes and mechanisms of aging elastomers. Firstly, the thermal decomposition process of unaged GAP elastomers under different atmospheres was investigated by TG-DSC (Figure 2).

Based on Figure 2 and Table 1, the thermal decomposition process of unaged GAP elastomers under different atmospheres all showed two stages. Under the nitrogen atmosphere (Figure 2a), the percentage weight loss of the elastomer was 34.87% in the first stage (190.13~283.58 °C), which was due to the breaking off of the -N_3_ groups on the molecular chain [24]. The weight-loss percentage in the second stage (283.61~518.63 °C) was 29.63%, which was mainly due to the decomposition of the urethane groups and the ether groups on the polymer chain [25], and there was still about 25% of residue remaining after the decomposition was complete. Meanwhile, the DSC curve only showed a clear exothermic peak in the temperature range of 190~300 °C, which coincided with the first weight-loss stage of the TG analysis, which was due to the exothermic production of N_2_ from the pyrolysis of -N_3_.

Compared with the nitrogen atmosphere, under air atmosphere (Figure 2b), the azide groups of the elastomer decomposed in advance, and the weight-loss percentage increased slightly. A small exothermic peak appeared in the DSC curve (170~200 °C), and the exothermic peak was at 188.32 °C. In the second stage, the weight-loss percentage increased dramatically, and the DSC curve showed an obvious exothermic peak in the temperature range of 485~680 °C. The elastomer backbone decomposition was more complete, and the residue content at the end of the thermal decomposition was very small. The sample was almost completely volatilized. This indicated that the oxygen atoms in the air interact with the chemical bonds of GAP elastomers to generate more free radicals and form oxidative pyrolysis, which allows the pyrolysis reaction to be more easily carried out and the decomposition to be more complete [26]. An oxidizing atmosphere promotes the decomposition of the GAP elastomer, and propellants stored in an air atmosphere are more susceptible to degradation reactions.

**Figure 3:**
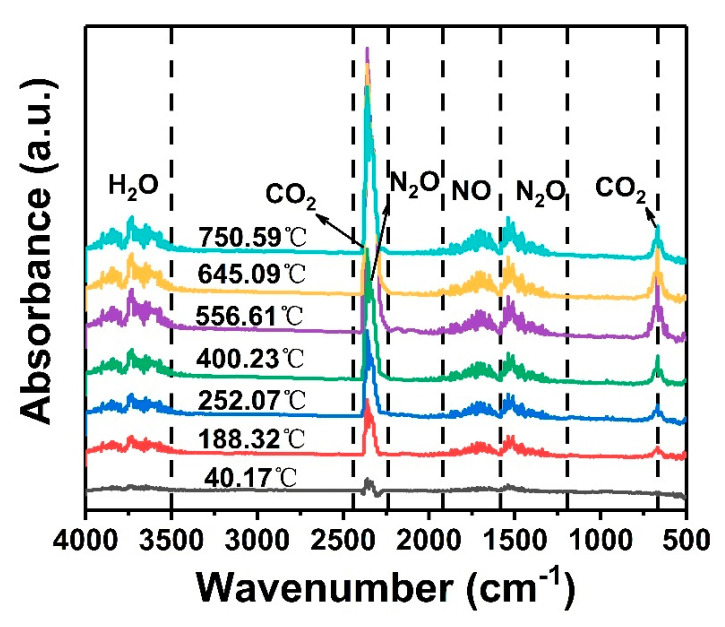
TG-IR curves of unaged GAP elastomers under air atmosphere.

Solid propellants are often stored in an air environment, and oxygen and other components in the air may initiate thermal oxidation reactions leading to the propellants’ decomposition and degradation. Therefore, the thermal decomposition of unaged GAP elastomers under air atmosphere was further analyzed by TG-FTIR (Figure 3). There is no peak in the spectrum at 40 °C, indicating that no gas escapes at room temperature. Thus, 40 °C was adopted as a reference temperature.

The main gaseous products of the thermal decomposition of GAP elastomers are N_2_O, NO, H_2_O, and CO_2_. The absorption peaks at 2270~2470 cm^−1^ and 550~760 cm^−1^ are related to CO_2_, while the absorption peaks at 2150~2270 cm^−1^ and 1400~1570 cm^−1^ are related to N_2_O [25]. In the initial stage of thermal decomposition (before 252 °C), the growth rate of NO and N_2_O gases was fast. With the increase in temperature, the absorption peaks of the gaseous products gradually increased, especially the absorption intensity of CO_2_. The absorbance of CO_2_ reached its maximum at 556.61 °C, and then the intensity decreased. This indicated that the GAP elastomer first cracked its azide groups in the air atmosphere. Although the molecular chain also began to depolymerize, the decomposition lagged slightly behind the breaking off of the -N_3_ groups [27]. With the increase in temperature, the GAP skeleton was oxidized and decomposed until it was complete [28].

### 3.2. Microstructure Evolution of GAP Elastomer during Aging

#### 3.2.1. Chemical-Structure Change

**Figure 4:**
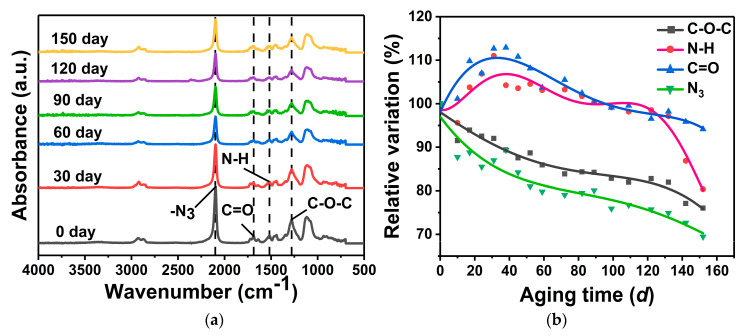
Changes in functional groups of the GAP elastomer with different aging times: (**a**) FTIR spectroscopic results; (**b**) Changes in the relative content of characteristic functional groups.

FITR was used to study the change in chemical composition of GAP elastomers during aging (Figure 4a). The peak at 1278 cm^−1^ corresponds to the stretching vibration peak of the ether bond (-C-O-C-), the peak at 1517 cm^−1^ corresponds to the vibration peak of the amine group (-NH) of the urethane group (-NHCOO-), the peak at 1689 cm^−1^ corresponds to the stretching peak of the carbonyl group (C=O) of the urethane group (-NHCOO-), and the peak at 2100 cm^−1^ is the asymmetric stretching vibration of the azide group (-N_3_) [29]. The peak absorption intensity of -N_3_, -C-O-C-, and -NHCOO- decreased with the increase in aging time. Cleavage of the azide group and breaking of the main chain occurred during thermal aging of the GAP elastomers.

The functional groups that did not change with aging time were selected as internal-standard reference peaks (ISRP). The methylene concentration did not change during the aging of the GAP elastomer, so the absorption peak at 1442 cm^−1^ (methylene) was used as the internal standard peak, and the absorbance of each functional group relative to the internal-standard group was calculated as a function of aging time. The relative absorbance was calculated from Equation (1) [23].

(1) Ai=AabsoluteiAISPRi

*A*_(i)_—relative absorbance;
*A_absolute(i_*_)_—absorbance of each functional group;
*A_ISPR(i)_*—absorbance of the internal-standard reference peak (methylene);
*i*—aging time.

The calculation results are shown in Figure 4b. During the aging process, the content of the functional groups -NH and C=O first increased and then decreased, which indicated that the post-curing reaction between molecular chains was dominant in the early stage of aging. With the extension of aging time, the post-curing reaction was completed, with the fracture of -NHCOO- as the main reaction, and the contents of -NH and C=O decreased. A downward trend was observed for -N_3_ and -C-O-C-, and their decomposition lasted throughout the whole thermal-aging process. After 150 *d* of aging, the relative content of -N_3_ decreased by 30.54%, that of -C-O-C- decreased by 23.97%, N-H decreased by 19.64%, and C=O decreased by 5.85%; -N_3_ showed the largest decrease. In the process of thermal aging, the azide groups of GAP elastomers broke rapidly, and the breaking of the polymer main chain was mainly due to the decomposition of ether bonds and urethane groups, which had similar thermal decomposition behavior.

**Figure 5:**
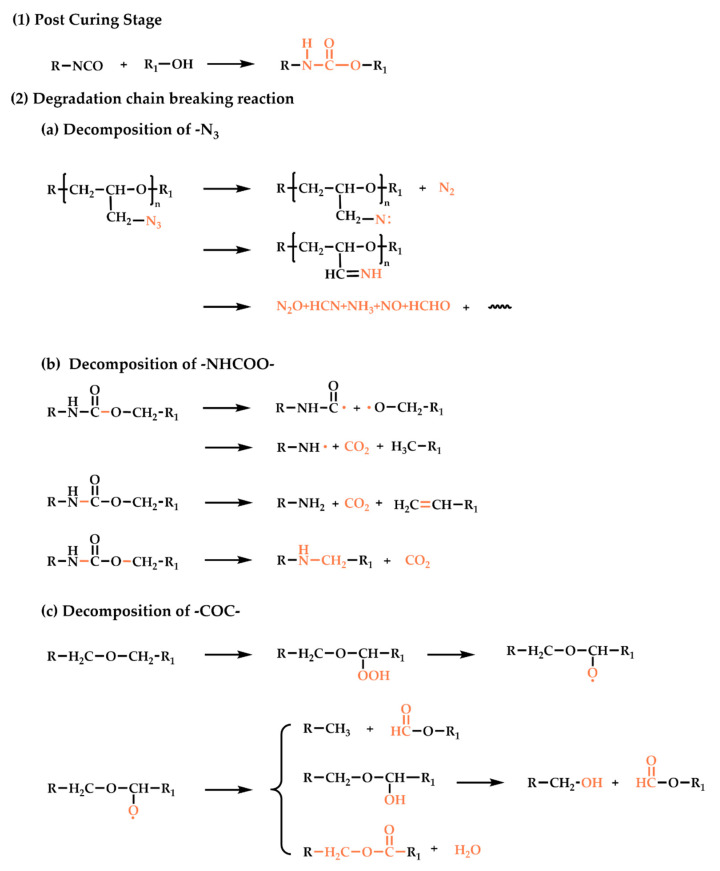
Reactions occurring during the thermal-aging process of the GAP elastomer.

During the aging process, the chemical-structure changes in the elastomers are mainly post-curing and degradation chain breaks. The chemical reaction is analyzed as follows, and the reactions that occur are summarized in Figure 5:

(1) Post-curing reaction.

Post-curing refers to the curing reaction that has not been completed during the normal curing cycle and continues slowly during storage. In the early stages of aging, due to the excess of the isocyanate curing agent in the elastomer (R = 1.4), the excess curing agent continues to react with the prepolymer during storage, and the post-curing reaction is mainly in the system.

(2) Degradation chain-breaking reaction.

As the weak links of the network structure, the -N_3_, -C-O-C-, and -NHCOO- functional groups are broken throughout the entire aging process.

    (a) Decomposition of -N_3_ groups.

During aging, the RN-N_2_ bonds of the GAP side group are broken to generate azine and release N_2_. The azine groups are highly reactive and can react with almost any C-H group of the neighboring molecules, and they can even be inserted into O-H or N-H bonds. At the same time, the generated azabine can also form imine structures through rearrangement, and then the imine recombines with the radical through H transfer to form NH_3_ or through C-C bond breaking to form products such as HCN [21].

    (b) Decomposition of -NHCOO- groups.

As the crosslinking points of the GAP bonding system, -NHCOO groups can react reversibly to form isocyanates and hydroxyl groups during aging. Secondly, the C-O bonds in the urethane groups are also easy to break, forming aminoformyl radicals and alkoxy radicals, and bonding with H on the β carbon to form carbamates; however, carbamates are unstable and continue to decompose into primary amines. At the same time, the C-O bonds connected to the carbamate also break, and the broken groups combine with -NH to form CO_2_ and secondary amines [30].

    (c) Decomposition of C-O-C- groups.

The -C-O-C- groups exist in the main chain of the elastomer. Oxidation of -C-O-C in the presence of light or heat during storage forms peroxides. The peroxide compound is extremely unstable and susceptible to oxygen-bond breakage, resulting in the formation of free radicals that trigger chain reactions leading to the production of various derivatives, including acids, alcohols, and esters. But free-radical crosslinking reactions can also be carried out, so that the crosslinking density increases [31].

#### 3.2.2. Changes in Network Structure

In the aging process, the change in the GAP elastomers’ network structure is the major reason for the impact on mechanical properties [32]. Here, the evolution of the crosslinked network caused by functional group changes was investigated by NMR. Due to the various chemical environments of the protons on the polymer chain, there are different transverse relaxation times (*T*_2_) under a constant magnetic-field intensity. The XLD model is used to fit and analyze the transverse relaxation time, which can be expressed as Equation (2) [33,34].

(2) $$M\left(t\right)=A\times exp\left(-\frac{t}{T_{20}}-\frac{1}{2}qM_{rl}\times t^{2}\right)+B\times exp\left(-\frac{t}{T_{21}}\right)+C\times exp\left(-\frac{t}{T_{22}}\right)$$

*M(t)*—attenuated signal;
*A*—proportions of crosslinking chains;
*B*—proportions of dangling chains;
*C*—proportions of free chains;
*T*_20_, *T*_21_, and *T*_22_—transverse relaxation time of these three chains;
*q*—anisotropy of the crosslinking chains;
*Mrl*—dipole moment below the glass-transition temperature.

**Figure 6:**
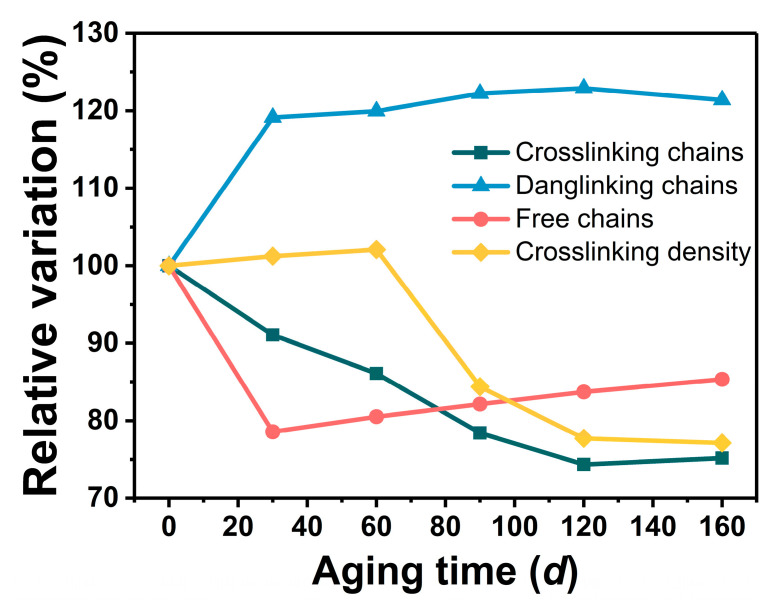
Change in GAP elastomer network structure with aging time.

For the process of thermal aging, the proportion changes in the three molecular chains are shown in Figure 6. The percentage of crosslinked chains (*P_c_*) in the unaged GAP elastomer that could effectively provide mechanical properties was relatively low (<25%), and most of it existed in the form of dangling chains (*P_d_*). During the aging process, the proportion of *P_c_* decreased gradually, while the proportion of free chains (*P_f_)* decreased sharply and then increased slowly, and the proportion of *P_d_* increased gradually. The change in crosslinking density showed a trend of increasing and then decreasing, and after 160 *d* of aging, the proportion of *P_c_* had decreased by about 24.82%, the proportion of *P_f_* had decreased by about 14.67%, and the proportion of *P_d_* had increased by about 21.38%.

In the early stage of aging, the curing agent and the hydroxyl group continued to react, and the crosslinking reaction was stronger than the degradation caused by chain breakage, so the crosslinking density of GAP elastomers at this stage increased slowly. The proportion of the crosslinked chains rose, and the proportion of the free chains decreased. In contrast, in the late stage of storage, breakage of the -NHCOO- and -C-O-C- groups dominated, resulting in a decrease in crosslink density and crosslinked chains, and the proportion of free chains increased. The proportion of dangling chains was related to the decomposition of the azide group, which increased as the azide group broke down during aging. The trends of the various molecular chain ratios with aging time were consistent with the FTIR results.

**Figure 7:**
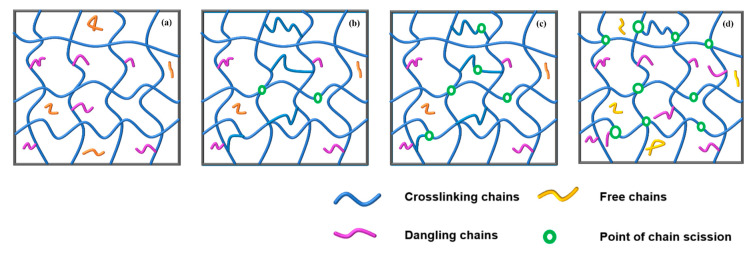
Conformational evolution of the crosslinked network structure of the GAP elastomer during the aging process: (**a**) Initial state, (**b**) stage 1, (**c**) stage 2, (**d**) stage 3.

By comprehensive analysis of the above experimental results, the microstructure of the GAP elastomer during thermal aging can be divided into three stages (Figure 7). The internal polymer network is complete before aging, as shown in Figure 7a. In the first stage, there is a fracturing of weak links in the network structure. Meanwhile, post-curing as the main reaction is still in progress, so the crosslinking point of the GAP elastomer is still increasing, as shown in Figure 7b. As aging progresses, molecular-chain-degradation breaks dominate, and the crosslinking density of the network structure begins to decrease (Figure 7c). In the later stages of aging (Figure 7d), the breakdown of molecular chains is still ongoing, and the crosslinked network of the GAP bonding system is severely damaged.

#### 3.2.3. Change in Activation Energy

According to Section 3.1, there were two reaction peaks in the DSC curve of GAP elastomers under air atmosphere. The second decomposition peak was the exothermic decomposition of the elastomer main chain. To further study the changes in the main chain structure of the GAP elastomer during the aging process, DSC tests were carried out on samples with different aging times, and the peak temperatures of the high-temperature thermal decomposition were obtained at different heating rates (10 K/min, 15 K/min, and 20 K/min). The kinetic parameters of the thermal decomposition were obtained by the Kissinger method.

The Kissinger method [35] is based on the displacement change in the peak temperature, where the peak temperature, *T_p_*, of the decomposition reaction is affected by the increase in the heating rate, and the activation energy is obtained by the inclination of the ln straight line. The method does not have to consider specific kinetic equations and is highly applicable and reproducible.

(3) $$\ln\left(\frac{\beta }{T_{P}^{2}}\right)=\ln\left(\frac{AR}{E_{a}}\right)-\frac{E_{a}}{RT_{p}}$$

The reaction rate corresponding to the peak temperature, *T_p_*, on the DSC curve is the maximum, and the derivative value at this peak temperature is zero, which can be obtained after collation:

(4) dln⁡β/Tpd1Tp=−EaR

*β*—the heating rate (K/min^−1^);
*T_p_*—the peak temperature of the DSC curve;
*A*—the pre-exponential factor;
*R*—the ideal gas constant (8.314 J/mol·K);
*E_a_*—the activation energy (kJ/mol).

The calculation results are shown in Table 2. In the pre-storage period, the oxidation activation energy of the samples did not change much. With the high-temperature aging, the thermal decomposition characteristics of the samples showed an obvious change trend, and the activation energy of the oxidation reaction showed a decreasing trend. In the samples aged for 160 *d*, the activation energy decreased by 25%. This suggested that the structure of the elastomer network did not change much during the pre-aging period, so the activation energy required for oxidative decomposition was unchanged. In the late aging period, molecular chain breakage was the main reaction, leading to the elastomer’s macromolecular backbone being fractured and the network structure being destroyed, so the energy required for the oxidative reaction was reduced, and it was more easily decomposed at high temperatures. The trend of activation energy for GAP elastomer backbone decomposition during thermal aging was consistent with the FITR and NMR results.

### 3.3. Change in Macroscopic Properties of the GAP Elastomer during Aging

#### 3.3.1. Macro-Morphological Changes

**Figure 8:**
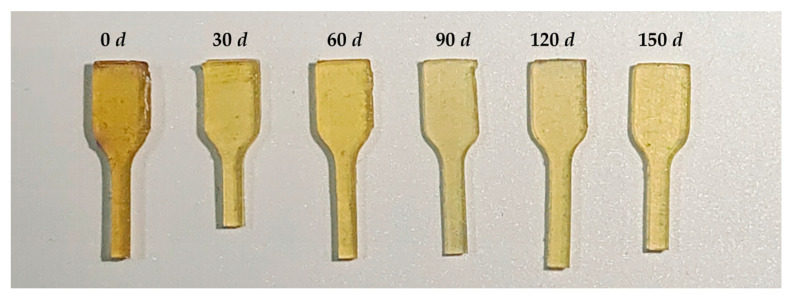
Changes in sample color during aging.

During thermally accelerated aging, the color of the GAP elastomer first gradually became lighter and then slowly turned yellow (Figure 8). This is because GAP contains a large number of azide groups, which is a color-generating group, so the GAP elastomer before aging is yellow. In the aging process, a large number of azide groups were in the decomposing elastomer, so the color of the elastomer gradually became lighter. With the aging process, the decomposition of the ether bonds and the urethane groups on the main chain increased, and the breakage of these groups generated unsaturated chrominance groups, so the color of GAP elastomer gradually deepened in the later storage period.

#### 3.3.2. Hardness Variation

**Figure 9:**
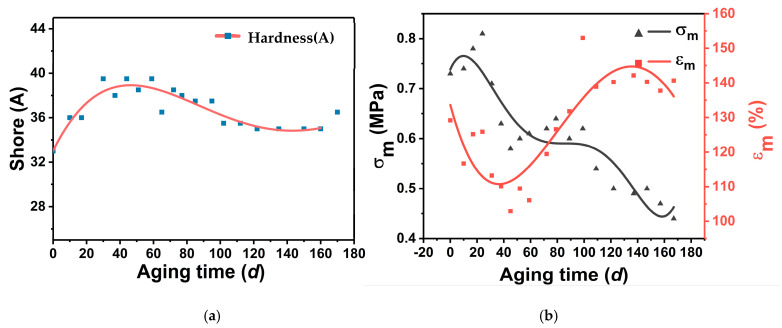
Changes in mechanical parameters with aging time: (**a**) Hardness-change curve; (**b**) Mechanical-change curve.

The relationship between surface hardness and time of GAP elastomers under thermally accelerated aging at 70 °C is shown in Figure 9a. With the prolongation of the aging time, the surface hardness of the GAP elastomers showed a trend of rising and then decreasing. The surface hardness did not change much at the late stage of aging. The change in hardness was mainly related to the network structure of the elastomer. The closer the network structure of the elastomer, the greater the hardness, while molecular chain breakage leads to a decrease in hardness. In the early stage of aging, the surface hardness of the GAP elastomer increased, indicating a post-curing reaction. The crosslinking degree of the GAP elastomer increased, forming a more dense network structure [36]. In the late stage of aging, the surface hardness of the GAP elastomer decreased, indicating that degradation of the chain-breaking reaction was dominant in the adhesive system, and the network structure of the macromolecule was destroyed.

#### 3.3.3. Mechanical Property Changes

Chemical aging has a significant effect on the mechanical properties of the elastomer. This is because aging affects the network structure of the elastomer, which is closely related to its mechanical properties [37]. If more crosslinking connections are formed in the crosslinking network, the mechanical properties show an increase in strength and a decrease in elongation; if the crosslinking network is destroyed, the mechanical properties show a decrease in strength and an increase in elongation [38].

During the aging process, the mechanical properties of the GAP elastomer also changed, and the changes in the mechanical parameters of the maximum tensile strength (σ_m_) and maximum elongation (ε_m_) are shown in Figure 9b. With thermally accelerated aging, the σ_m_ showed a tendency to increase and then decrease, while the ε_m_ first decreased, then rose and subsequently decreased again. In the early stage of aging, the σ_m_ rose sharply and the ε_m_ decreased rapidly due to the post-curing reaction, and the network structure of the elastomer was denser. As aging proceeded, the σ_m_ of the elastomer decreased, while the ε_m_ rose and then decreased slowly. This indicated that with the aging process, the post-curing reaction had been completed, and the degradation of GAP elastomer chain breaking was stronger than the crosslinking effect. The -NHCOO- and-C-O-C- groups within the molecular chains were broken, and the degree of crosslinking between the network was reduced. With the degradation of the broken chain continuing, the network structure was severely damaged, and the ε_m_ also began to fall. The change in mechanical properties was consistent with the change in the microstructure, which was also divided into three stages.

The mechanical properties of GAP elastomers are the decisive factors for the mechanical properties of propellants. According to the mechanical property changes in GAP elastomers, it can be hypothesized that GAP propellants will likewise show similar changes during the storage process. In the early stage of aging, the propellant σ_m_ rises and the ε_m_ decreases due to post-curing, but after the aging period, the σ_m_ and ε_m_ are in a decreasing trend, and the failure of GAP propellants is mainly manifested in the maximum tensile strength.

## 4. Conclusions

In this work, changes in the macroscopic properties and microstructure of GAP elastomers during thermal aging were investigated. It was found that the breakage of azide, urethane, and ether bonds occurred throughout the whole aging process of GAP elastomers, and the crosslinking density increased firstly and then decreased. During the aging process, the mechanical property changes in GAP elastomers were consistent with the microstructure changes, showing the characteristics of segmental changes. The elastomeric showed three stages in the aging process: The first stage was dominated by post-curing, and the crosslinked network was denser. Then, the crosslinked network was destroyed by the degradation and chain-breaking main reaction. In the third stage, the degradation chain-breaking continued, and the crosslinked network was severely damaged. This study is expected to promote a deeper understanding of the aging mechanism of GAP elastomers and provide guidance for improving the storage life of GAP propellants.

## Figures and Tables

**Table 1:** TG-DSC data of unaged GAP elastomers under different atmospheres.

	Low-Temperature Decomposition Stage				High-Temperature Decomposition Stage			
	*T_i_* (°C)	*T_TD_* (°C)	*T_e_* (°C)	*M_S_* (%)	*T_i_* (°C)	*T_TD_* (°C)	*T_e_* (°C)	*M_S_* (%)
N_2_	190.13	253.51	283.58	34.87	283.61	/	518.57	29.63
O_2_	172.73	252.41	285.26	39.28	293.77	556.61	685.93	59.58

*T_i_*, the onset temperature of pyrolysis; *T_TD_*, the peak temperature of pyrolysis; *T_e_*, the onset temperature of the end of pyrolysis; *M_S_*, weight loss in heat.

**Table 2:** Kinetic parameters of the second reaction peak of the thermal decomposition of GAP elastomers under air atmosphere.

	Heating Rate	0	30	60	90	120	160
Aging Time (*d*)							
10 K/min		557.28	560.23	558.62	560.60	558.67	556.45
15 K/min		577.66	577.42	578.37	580.34	579.74	583.70
20 K/min		588.63	592.29	592.07	598.84	599.12	600.24
Ea (kJ/mol)		115.53	115.86	109.64	95.61	89.46	80.39
A (min^−1^)		3686.17	3686.17	1459.76	163.85	65.26	16.06

## Data Availability

Data are contained within the article.
